# Platelet Phenotyping and Function Testing in Thrombocytopenia

**DOI:** 10.3390/jcm10051114

**Published:** 2021-03-07

**Authors:** Kerstin Jurk, Yavar Shiravand

**Affiliations:** 1 Center for Thrombosis and Hemostasis (CTH), University Medical Center of the Johannes Gutenberg University Mainz, 55131 Mainz, Germany; 2 Department of Molecular Medicine and Medical Biotechnology, University of Naples Federico II, 80131 Naples, Italy; shiravandy@gmail.com

**Keywords:** thrombocytopenia, bleeding, platelet function tests, platelet disorders, platelet count, flow cytometry

## Abstract

Patients who suffer from inherited or acquired thrombocytopenia can be also affected by platelet function defects, which potentially increase the risk of severe and life-threatening bleeding complications. A plethora of tests and assays for platelet phenotyping and function analysis are available, which are, in part, feasible in clinical practice due to adequate point-of-care qualities. However, most of them are time-consuming, require experienced and skilled personnel for platelet handling and processing, and are therefore well-established only in specialized laboratories. This review summarizes major indications, methods/assays for platelet phenotyping, and in vitro function testing in blood samples with reduced platelet count in relation to their clinical practicability. In addition, the diagnostic significance, difficulties, and challenges of selected tests to evaluate the hemostatic capacity and specific defects of platelets with reduced number are addressed.

## 1. Introduction

Platelet bleeding disorders are a heterogeneous group in terms of frequency and bleeding severity. They are characterized by qualitative/function and/or quantitative/number platelet defects. Patients with a platelet count of less than 150 × 10^9^/L in whole blood present with thrombocytopenia, which is caused by inadequate megakaryopoiesis and platelet production or enhanced platelet clearance due to platelet destruction or pathological platelet activation. Rare gene defects cause inherited thrombocytopenia, but thrombocytopenia is more frequently acquired in response to systemic disease manifestations (e.g., uremia, liver disease, and myeloproliferative disorders), autoimmune diseases (e.g., idiopathic thrombocytopenic purpura (ITP), heparin-induced thrombocytopenia (HIT), thrombotic thrombocytopenic purpura (TTP), and antiphospholipid syndrome), infection, pregnancy, surgery, transfusion, and certain drugs (e.g., heparin, thiazide diuretics, and tamoxifen). Severe thrombocytopenia with a platelet count of less than 50 × 10^9^/L increases the risk of spontaneous skin and mucocutaneous bleeding diathesis, such as petechiae, ecchymoses, epistaxis, menorrhagia, and gastrointestinal bleeding. However, bleeding due to major hemostatic challenges (e.g., surgery and trauma) is especially frequently observed when moderate (50–100 × 10^9^ platelets/L) and even mild thrombocytopenia (>100–<150 × 10^9^ platelets/L) is associated with inherited or acquired platelet dysfunction [1].

Though inherited platelet function disorders are rare, their prevalence is underestimated due to limited diagnostic potential. Multiple platelet phenotype and functional tests are recommended by different laboratory guidelines for the diagnosis of platelet function disorders [2,3,4]. Recently, the Scientific and Standardization Committee (SSC) “Platelet Physiology” of the International Society on Thrombosis and Haemostasis (ISTH) established a three-step algorithm of platelet function tests for the identification of well-known and complex entities of inherited platelet function disorders, which are commonly associated with mild-to-moderate bleeding diathesis [3,5]. However, only a couple of tests allow for a standardized and easy-to-use analysis of platelet function in clinical routine practice. Indeed, most platelet function tests require skilled personnel and are restricted to specialized platelet laboratories, which have to ensure intra-laboratory standardization and validated reference values. Furthermore, platelet function testing in thrombocytopenia requires expertise and experience about sensitivity and the limitations of reduced platelet counts for each test. This review addresses major indications, methods/assays for platelet phenotyping, and in vitro function testing in blood samples with reduced platelet count in relation to their clinical practicability. In addition, the diagnostic significance, difficulties, and challenges of selected tests to evaluate the hemostatic capacity and specific defects of platelets in reduced number are summarized. 

## 2. Indications for Testing Platelet Phenotype and Function in Thrombocytopenia

Though a low platelet count significantly increases the risk for hemorrhagic complications, many patients with thrombocytopenia, even with a severe reduction of the platelet count, do not suffer from spontaneous, clinically relevant bleeding. Previous studies have not provided much evidence that bleeding risk is associated with platelet count in patients with acquired thrombocytopenia caused by hematological malignancies, non-hematological cancer, sepsis, and chronic liver and renal disease [6,7,8], and impaired platelet function has been demonstrated in these disease entities [9,10,11,12]. However, the molecular mechanisms responsible for platelet dysfunction in acquired thrombocytopenias are only marginally understood. There is evidence that alterations in megakaryopoiesis and platelet production leads to defective platelet activation/aggregation and granule storage pool deficiency (SPD) in patients with acute leukemia and myelodysplastic syndromes [9]. Patients with advanced chronic liver, e.g., cirrhosis, and kidney disease, e.g., uremia, are known to show impaired platelet activation induced by several agonists, as determined by flow cytometry (see Section 5.3) and aggregation tests (see Section 5.1). Acquired storage pool defects, defective transmembrane signaling associated with the reduced surface expression of integrin αIIbβ3 and proteolytically cleaved glycoprotein (GP) Ibα, dysregulated arachidonic and thromboxane A_2_ (TxA_2_) metabolism, diminished cytosolic Ca^2+^ release/entry, and elevated cytosolic levels of inhibitory second messengers, i.e., cyclic adenosine monophosphate (cAMP) and guanosine monophosphate (cGMP), have been observed for both systemic diseases. On the one hand, it has been suggested that the hyporeactive platelet phenotype in vitro is caused by in vivo activation and “exhaustion” during intravascular activation. On the other hand, increased levels of different plasma factors, e.g., fibrin(ogen) degradation products, bile salts, ethanol, and uremic toxins (like phenol, phenolic acid, and guanidine succinic acid), may interfere with platelets, thus leading to hyporeactivity [10,12,13]. An increased in vivo activation status of platelets, leading to platelet exhaustion and impaired platelet reactivity in vitro, is observed in patients with sepsis when bacterial and viral infections are dysregulated. Here, platelet activation is mediated by direct interactions with pathogens via pattern recognition receptors, the release of inflammatory mediators from leukocytes and endothelial cells, and complement activation [11]. The enhanced formation of pathogen-coated and -stimulated platelets drives platelet clearance and turnover, leading to thrombocytopenia and increased levels of more reactive immature/reticulated platelets, respectively (see Section 3.3). The immature platelet fraction has been suggested to predict sepsis severity [14].

In addition, platelet dysfunction associated with acquired thrombocytopenia is frequently observed in hospitalized patients to be caused by iatrogenic etiologies, including drug-induced immune thrombocytopenia [15,16], major surgery, and extracorporeal/mechanical circulatory support. Ventricular assist devices (VADs) and the extracorporeal membrane oxygenation (ECMO) device induce pathological hemodynamics associated with high shear stress and contact of platelets with non-physiological mechanical surfaces. Acquired von Willebrand disease (VWD) may result from elevated shear stress conditions and is characterized by the loss of high-molecular-weight multimers of the von Willebrand factor (VWF), leading to impaired VWF-mediated platelet functions. Furthermore, platelets become activated by interacting with non-physiological mechanical surfaces, leading to the pronounced platelet consumption and impaired capacity of granule secretion and aggregation in vitro [17]. 

**Figure 1:**
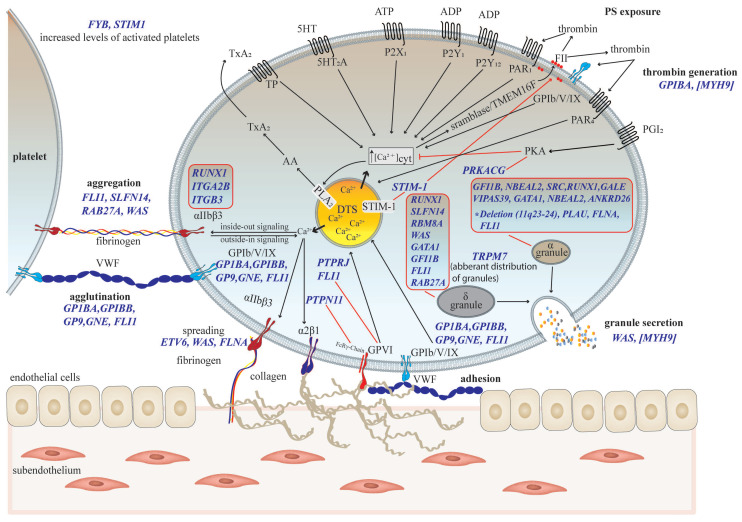
Current view of important thrombocytopenia-causing genes associated with platelet function defects. Gene names (blue italic font), activating responses (black arrow), inhibitory effects (red blunted arrow), and gene mutation consequences (red line) are indicated, and further details are listed in Table S1 [18,19,20,21,22]. *MYH9*-associated platelet dysfunction according to a case report [23]. VWF: Von Willebrand factor; ADP: adenosine diphosphate; PAR: proteinase-activated receptor; PLA_2_: phospholipase A_2_; PKA: protein kinase A; STIM-1: stromal interaction molecule-1; TMEM16F: transmembrane protein 16F; TxA_2_: thromboxane A_2_; TP: TxA_2_ receptor; AA: arachidonic acid; PGI_2_: prostacyclin; DTS: dense tubular system; GP: glycoprotein; PS: phosphatidylserine.

Inherited thrombocytopenias are commonly caused by pathogenic variants of genes encoding for proteins involved in megakaryopoiesis (e.g., thrombopoietin (THPO)/ myeloproliferative leukemia virus (MPL) signaling and transcription regulation), megakaryocyte maturation (i.e., granulopoiesis and trafficking), and platelet production/release (i.e., cytoskeleton regulation and glycoprotein receptor signaling). For a detailed overview of inherited thrombocytopenias, the reader is referred to the review about inherited thrombocytopenias by Paolo Gresele in this special issue on “The Latest Clinical Advances of Thrombocytopenia.” Recent studies have shown that a significant proportion of defective genes causing thrombocytopenia also affect platelet function. Figure 1 and Table S1 present an overview of important disease-causing genes related to thrombocytopenia that are associated with platelet function defects. Biallelic Bernard–Soulier syndrome (BSS), gray platelet syndrome (GPS), platelet type VWD, and Wiskott–Aldrich syndrome (WAS) are well-known platelet number and function disorders that are additionally characterized by abnormal platelet volume. Thus, the intrinsic hemostatic capacity of platelets needs to be viewed as reflection of platelet count, size/volume (platelet mass), and function. 

Overall, platelet function testing in thrombocytopenia is essential (1) if the level of platelet count reduction cannot explain the bleeding tendency and (2) to complement the diagnosis of known and novel thrombocytopenic diseases with potential bleeding risk, even when pathogenic or likely-pathogenic mutation(s) could be identified by next generation sequencing (NGS). Platelet function tests allow for, depending on the technique, (1) the evaluation of the overall hemostatic platelet activity and (2) the identification of specific platelet function defects and their molecular targets. It is noteworthy to recapitulate the strengths and limitations of each platelet function test with regard to the sensitivity and specificity for different platelet function parameters and their utility for the analysis of the global hemostatic capacity of whole blood, the intrinsic hemostatic capacity of platelets, and specific platelet responses/molecular targets. Platelet function testing may only contribute to the evaluation of the potential bleeding risk in thrombocytopenia, as other hemostatic factors of other blood and vascular cells could impair overall hemostatic capacity. In addition, prospective clinical cohort studies, which are yet very limited, are necessary for the evaluation of the predictive value of platelet function tests for bleeding, especially in case of thrombocytopenic diseases. Thus far, there is a lack of evidence for most platelet function assays to predict prophylactic platelet transfusions or other “pro”-platelet therapies. 

## 3. Platelet Phenotyping in Thrombocytopenia

Before platelet function testing should be applied, an extensive clinical evaluation of the patient’s and family’s bleeding history; an examination of other disease manifestations, medication and/or food affecting platelet function; a laboratory assessment of platelet count, size, morphology, as well as of plasma coagulation/fibrinolysis and VWF parameters, are essential. 

### 3.1. Bleeding Assessment Tool (BAT)

The use of a standardized bleeding assessment tool (BAT) is recommended to calculate a bleeding score for the accurate evaluation of the bleeding tendency. In addition to the bleeding score of the World Health Organization (WHO), several BATs have been established, especially for type 1 VWD [24]. More recently, the SSC “Platelet Physiology” of the ISTH performed a validation study to test the diagnostic utility of the type 1 VWD ISTH-BAT for inherited platelet function and number disorders in a large cohort with more than of 1000 patients across 17 countries worldwide [25]. Inherited platelet function disorders (IPFDs), including major disorders with 40% Glanzmann thrombasthenia, 11% δ-SPD, and about 10% primary secretion defect, as well as 10% thrombocytopenic patients with biallelic BSS, showed a median bleeding score of 9 and an excellent discrimination power between IPFD (bleeding score > 6) and age/sex-matched healthy controls compared to the WHO bleeding score. In contrast, inherited platelet number disorders (IPNDs), including major disorders with about 41% *MYH9*-related disorders, 22% *ANKRD26*-related thrombocytopenia, 19% monoallelic BSS, and 8% *ETV6*-related thrombocytopenia, had a median bleeding score of 2, and ISTH-BAT had a poor discrimination power between IPND and healthy controls. This study also confirmed that patients with IPFD have a higher number of bleeding symptoms and a higher percentage with clinically relevant symptoms than patients with IPND. However, a specific comparison of the ISTH bleeding score between thrombocytopenic patients without and with associated platelet dysfunction has not been performed yet.

### 3.2. Laboratory Assessment of Platelet Count, Size and Global Morphology

Platelet counting in ethylenediaminetetraacetic acid (EDTA)-anticoagulated whole blood is the first step of platelet phenotyping, especially to exclude thrombocytopenia. Automatic impedance-based cell counters enable the discrimination of low platelet counts when the mean platelet volume is calculated within normal ranges. However, enlarged or giant platelets are not detected as platelets, and sometimes conjugation between platelets and with leukocytes occur (pseudo-thrombocytopenia in EDTA-anticoagulated whole blood), leading to artificially lower platelet counts. In case of an automatically calculated reduced platelet count, the analysis of a May–Grünwald–Giemsa-stained blood smear of EDTA- and non-EDTA- (e.g., 3.2%/109 mM tri-sodium-citrate) anticoagulated whole blood is recommended by light microscopy for the calculation of the count of normal and abnormal sized platelets (e.g., microplatelets detected in WAS and giant platelets observed in biallelic BSS), as well as to exclude pseudo-thrombocytopenia. This method also allows for the assessment of the first morphological alterations of platelets. A pale phenotype of large platelets is indicative of a deficiency of α-granules, as observed for the classical GPS caused by pathogenic *NBEAL-2*-variants, *GFI1b*-related thrombocytopenia caused by *GFI1b*-mutations, and the arthrogryposis—renal dysfunction—cholestasis (ARC)-syndrome caused by pathogenic gene variants in *VPS33B* or *VIPAS39*. *GATA1*-related diseases are typically characterized by a subpopulation of large and vacuolated platelets, and they could be associated with a decreased number of α-granules or a combined α-/δ-storage pool defect [26,27]. In contrast, alterations in the phenotype of neutrophils with “Döhle-like” bodies associated with giant platelet populations are typical for *MYH9*-related diseases such as the May–Hegglin anomaly [18,28] (Table S1 and Figure 1). However, platelet δ-granule defects are not detectable on a conventionally stained blood smear.

### 3.3. Blood Smear Analysis by Immunofluorescence Microscopy 

Recently, advanced immunofluorescence microscopy was used to analyze dried blood films stained with fluorochrome-labeled monoclonal antibodies detecting platelet-specific, surface receptor-related proteins (e.g., GPIb and GPIX of the VWF-receptor complex), α-granule membrane and cargo proteins (P-selectin, thrombospondin-1 (TSP-1), and VWF), and cytoskeletal proteins (i.e., β1-tubulin and filamin-A). This technique facilitates the phenotypic diagnosis of a BSS, α-granule deficiency-related macrothrombocytopenias (e.g., GPS and *GFBI1*-related thrombocytopenia), and *TUBB1*- and *FLNA*-related thrombocytopenia [29,30] (Table S1 and Figure 1). 

### 3.4. Quantitation of Immature/Reticulated Platelets

On the other hand, increased thrombopoiesis in acquired thrombocytopenia, as observed in immune thrombocytopenia or after severe infection, leads to an increased number of young/immature circulating platelets that are larger and enriched in messenger RNA—so-called “reticulated” platelets [31]. There is evidence that reticulated platelets are more reactive than “mature” platelets, and reticulated platelets have been suggested to have the potential to partly compensate for impaired hemostasis in thrombocytopenic ITP patients [32]. However, the use of immature platelet levels as predictive markers for bleeding in thrombocytopenic patients needs further validation in large prospective clinical studies. Some types of fully automated hematology analyzers used in clinical routine laboratories are based on light scatter and fluorescence detection, which allows for the quantification of large(r) and reticulated platelets stained with RNA-specific fluorescent dyes (immature platelet count or fraction in percent). Though automated hematology analyzers offer standardization for different labs, results obtained from laboratories of different companies are not yet comparable [31]. 

### 3.5. Flow Cytometry

The flow cytometric analysis of platelet count, size, and immature platelets (RNA stained by thiazole orange or SYTO-13) serves as alternative technique, but skilled personnel is required to permit intra-laboratory standardization and validation [33,34]. The quantitation of platelet surface receptors in citrated whole blood or the assessment of mepacrine uptake in isolated platelets ex vivo at resting conditions by flow cytometry is recommended to support the diagnosis of receptor-linked thrombocytopenias (e.g., BSS) [3] or δ-SPD [35] (see Section 5.3). 

### 3.6. Phenotyping of Platelet Granule Defects by Electron Microscopy and ELISA

Sophisticated transmission electron microscopy techniques allow for the detailed analysis of the altered ultrastructure and morphology of platelets, even at low platelet count [36,37]. In specialized laboratories, a reduced number or deficiency of platelet α- and δ-granules can be validated by transmission electron microscopy and whole mount electron microscopy, respectively [38]. Advanced focused ion beam-scanning electron microscopy enables the visualization of the spatial distribution of platelet α- and δ-granules but is also restricted to specialized laboratories [39]. Commercially available ELISA-based tests allow for the quantitation of specific α-granule proteins, e.g., platelet factor 4 (PF4), β-thromboglobulin, and TSP-1, in the lysates of resting platelets, which are separated from plasma proteins. The sensitivity of such ELISAs determines the cut-off for the platelet count in isolated platelet samples. Granule defects are frequently observed for distinct syndromic thrombocytopenias such as WAS, GPS, and ARC (reduced number or deficiency of α-granules), as well as Paris-Trousseau and Jacobsen syndromes (alterations in α-granule phenotype and δ-SPD) (Table S1 and Figure 1).

## 4. Point-of-Care-Related Platelet Function Tests

The practicability of platelet function tests in clinical routine is defined by point-of-care (POC) attributes, e.g., near patient usage, simple and easy use, automatization, and rapid read outs. These attributes prefer analysis in whole blood without sample processing. The spectrum of platelet function tests is remarkable [40]. However, only a few tests share the POC criteria. This section summarizes the limitations and challenges of thrombocytopenia for a selection of frequently clinically used platelet function tests. 

### 4.1. Impedance-Based Aggregometry—Multiplate^®^ Analyzer

Platelet aggregation responses induced by a variety of platelet agonists are widely used read-out parameters for screening platelet function in primary hemostasis (Figure 1). Impedance-based multiple electrode aggregometers allow for the quantitation of platelet aggregation in anticoagulated whole blood through the detection of increased electrode coating by aggregated platelets over time under stirring conditions at 37 °C. These global devices allow for the assessment of the hemostatic capacity of platelets determined by platelet count, size, and function, but they also represent the overall hemostatic capacity of whole blood, which is further affected by red blood cells (hematocrit) and leukocytes (count and function) [2,41]. 

The Multiplate analyzer^®^ serves as clinical pioneer of multiple electrode aggregometry, which was originally developed as semi-automated POC monitoring test for antiplatelet drugs (e.g., aspirin, purinergic adenosine diphosphate (ADP) receptor P2Y_12_ blockers, and GPIIb/IIIa antagonists) in diluted hirudin-anticoagulated whole blood [42,43]. However, its utility for the diagnosis of platelet dysfunction is quite limited. This test showed a good correlation with light transmission aggregometry according to Born (see Section 5.1) in detecting impaired platelet aggregation from patients with severe platelet function disorders and normal platelet count, such as Glanzmann thrombasthenia [44,45]. Conversely, it lacks sensitivity and specificity for the diagnosing of mild platelet disorders [46,47], which are frequently observed in thrombocytopenias [18] (Table S1). In addition, multiple electrode aggregometry shows a high sensitivity for platelet count in samples from thrombocytopenic patients [48], as well as from healthy donors after the adjustment of platelets in vitro to low and even to normal (150–450 × 10^9^/L) counts [49]. Notably, the platelet count is recommended to be adjusted with a buffer (e.g., Tyrode’s buffer at pH 7.4) and not with platelet-poor plasma (PPP) due to the potential inhibitory effects of PPP on platelet function [49,50]. Thus, the Multiplate device is able to screen the hemostatic capacity of the whole blood environment and antiplatelet drugs, but it is not recommended for the specific detection of platelet dysfunction in thrombocytopenia [3,49]. Alternatively, control samples with adjusted platelet count have the potential to serve as references [32,49,51]. However, the preparation of such standardized and validated control samples is very time-consuming and requires expertise in platelet handling, which is commonly not feasible in clinical practice.

### 4.2. Platelet Function Analyzer (PFA)

The platelet function analyzer (PFA-100^®^, INNOVANCE^®^ PFA-200) was originally developed as rapid and standardized technique to assess bleeding time in vitro. This test measured the occlusion time in seconds of a collagen/ADP or collagen/epinephrine-coated aperture when platelets adhere and aggregate under high arterial shear rate (>5000/s) in buffered citrated (3.8%) whole blood [52]. Thus, global VWF-dependent primary hemostasis is screened, and the pathological prolongation of the closure time may be indicative of a moderate-to-severe VWD or platelet function defects [47,53,54] but not for mild platelet disorders (e.g., storage pool defects and primary secretion defects) due to the lack of specificity and sensitivity [55,56]. In addition to VWF levels, important determinants are low hematocrit and platelet count. Therefore, samples from thrombocytopenic patients with a platelet count <100 × 10^9^/L and/or a hematocrit measurement of less than 30% should not be analyzed. Due to all these limitations, the PFA device is no longer recommended by the ISTH and others for the diagnosis of platelet function defects [3,57].

### 4.3. Impact-R™ System

The Impact-R™ system represents a semi-automated cone and plate(let) analyzer (CPA). Platelet adhesion and aggregation are determined on a polystyrene surface immobilized by plasma proteins (e.g., VWF) in citrated whole blood under arterial shear rate (1800/s), which is induced by a rotating cone. May–Grünwald-stained platelets are quantified as the percent surface coverage and average size in µm by an image analyzer [58]. Similar to the PFA device, the CPA is sensitive to VWF plasma levels, hematocrit, and platelet count, but it could be used as a supportive platelet function test for the diagnosis of biallelic BSS [59] and for the analysis of the effect of low plasma a disintegrin and metalloprotease with thrombospondin type 1 motif 13 (ADAMTS-13) activity on VWF-mediated platelet adhesion and aggregation in patients with TTP [60]. The CPA device even allows for the analysis of blood samples from patients with severe acquired thrombocytopenia such as ITP [61]. However, previous studies with thrombocytopenic patients have been very limited and did not adjust for platelet count and hematocrit in control samples [62].

### 4.4. Thromboelastography/Metry

The viscoelastic-based methods, thromboelastography (TEG^®^) and thromboelastometry (ROTEM^®^) quantify different parameters of global coagulation and fibrinolysis, respectively, in anticoagulated whole blood, characterized by clot initiation, formation, strength/stiffness, and clot resolution/lysis. Both devices were primarily developed for the peri-/post-operative coagulation and transfusion management [63]. Platelet reactivity in vitro significantly contributes to thrombin generation, clot retraction, and the activation of fibrinolysis [64,65], and the interaction between platelets, fibrin, and its stabilization by activated factor XIII are important determinants for clot strength. However, thromboelastography/metry is not sufficiently sensitive to alterations in platelet function induced by antiplatelet drugs such as aspirin and P2Y_12_ blockers [66]. Therefore, the TEG system has been updated to the Platelet Mapping™ system, which is more sensitive to the platelet-induced increase of clot strength in response to arachidonic acid and ADP. However, sensitivity to platelet dysfunction is still limited and only valid for severe platelet function defects, as observed for the non-thrombocytopenic inherited disorder Glanzmann thrombasthenia [67]. Strikingly, clot strength is also affected by low platelet count in thrombocytopenic samples, so it is not surprising that a lower clot strength is observed in samples from severe and moderate thrombocytopenic patients with BSS and immune thrombocytopenia [67,68,69]. A prospective observational study, ATHENA (risk factors for bleeding in haematology patients with low platelet counts), including 50 patients with hematological malignancies and moderate-to-severe thrombocytopenia, showed a significant association between the ROTEM parameter “maximum clot firmness (MCF)” and bleeding among these patients, which was lost after the statistical adjustment for platelet count [70]. This study demonstrated that the observed low MCF exclusively depends on a low platelet count and was not further affected by potentially additional platelet dysfunction. Thus, TEG and ROTEM devices quantify the overall coagulation and fibrinolysis capacity of blood, but they cannot discriminate between the effects of platelet count and function and therefore should not be used for the diagnosis of platelet function defects in thrombocytopenic samples [66,71]. 

In summary, most of the clinically used whole blood platelet function tests, which share POC attributes, are recommended to screen global processes of primary and secondary hemostasis. These overall hemostatic activities depend on platelet function, count, and size/volume—in addition to hematocrit, leukocyte count/function and plasma coagulation/fibrinolysis factors—and are not sensitive to evaluate platelet function defects in samples with low platelet counts. The establishment of standardized control samples with adjusted platelet count or platelet mass may solve this limitation, but that requires skilled personnel and represents a great challenge for clinical routine laboratories. The impact of thrombocytopenia on POC-related platelet function tests, described here, is summarized in Table S2. 

## 5. Specialized Platelet Function Tests

Light transmission aggregometry according to Born [72] still represents the gold standard test for platelet function, as well as for the diagnosis of platelet function disorders in clinical routine laboratories. In addition, the lumi-aggregometry has also gained more and more clinical feasibility for the diagnosis of defects in platelet δ-granule secretion, which are associated with inherited (Figure 1) and acquired thrombocytopenias. Though LTA and lumi-aggregometry are being introduced more and more in clinical centers, they require skilled personnel and time-consuming standardization, which is already established in specialized platelet laboratories. Additional important assays predominantly developed for research proposes have been implemented in a panel of tests for the diagnosis of platelet function disorders.

Specific tests allowing for platelet function analysis in anticoagulated whole blood are preferable if only small blood volumes from patients are available or if specific platelet functions require testing in a more physiological environment. For the analysis of platelet-specific activation responses, platelets are isolated as platelet-rich plasma (PRP) or depleted from plasma proteins and leukocytes by advanced washing procedures [73] or gel-filtration [74], which also allow for a concentration process for platelets and thereby platelet analysis for patients with severe thrombocytopenia. The latter techniques are time-consuming and require skilled personnel with extensive expertise in platelet handling and processing.

This section focuses on advantages, options, and clinical utility, as well as the limitations and challenges, of specialized tests for the evaluation of platelet function defects from thrombocytopenic patients. Table S2 summarizes the impact of thrombocytopenia on specialized platelet function tests, as described in the following sections. 

### 5.1. Light Transmission Aggregometry in Platelet-Rich Plasma (LTA)

The turbidimetric-based LTA according to Born [72] photometrically detects an increase in light transmittance over time at 37 °C, which results from the agonist-induced aggregation (fibrinogen-dependent) or agglutination (VWF-dependent) of stirred platelets in platelet-rich plasma. In contrast to impedance-based aggregometry, LTA requires a centrifugation step of citrated whole blood to obtain PRP and a further centrifugation step of the buffy coat to obtain PPP, which serves as a reference [75]. In addition, LTA allows for a more precise analysis of the platelet aggregation process, which includes shape change properties and the detection of reversible platelet aggregation (disaggregation) indicative of a used threshold agonist concentrations and for defects in the platelet release of amplification agonists such as ADP/ATP from the δ-granules and TxA_2_. 

Multiple platelet agonists are commercially available to evaluate platelet aggregability in vitro. The SSC “platelet physiology” of the ISTH recommends LTA as a first-line screening assay for the diagnosis of inherited platelet function disorders by using classical platelet agonists, e.g., ADP (targeting the purinergic ADP receptors P2Y_1_ and P2Y_12_), arachidonic acid (targeting Cox-1-related pathways for synthesis and release of TxA2 and TxA2 receptor (TP receptor)), collagen (targeting the collagen receptors integrin α2β1 and GPVI), epinephrine (targeting α2A receptor), and ristocetin (targeting VWF-mediated crosslinking of platelets via the GPIb/V/IX receptor complex (agglutination)) [3]. Further platelet agonists are recommended for extended analysis, including selective GPVI agonists, e.g., collagen-related peptide (cross-linked collagen-related peptide (CRP-XL)) and convulxin), α-thrombin (targeting the thrombin receptors GPIbα, proteinase-activated receptor (PAR)-1, and PAR-4), PAR-1 activating peptide (i.e., thrombin receptor activating peptide-6 (TRAP-6)), PAR-4 activating peptide, TxA2 mimetics (e.g., U46619), Ca^2+^-ionophore, and phorbol 12-myristate 13-acetate (PMA). International guidelines refer to pre-analytical considerations and pitfalls, as well as standardized procedures, which also include recommendations for standardized agonist concentrations [76,77]. 

Recently, an inter-laboratory external quality assessment was performed by the THROMKID-Plus Study Group of the German Society of Thrombosis and Haemostais Reasearch (GTH) and German Society for Pediatric Oncology and Hematology (GPOH) in Germany and Austria for the standardization of light transmission aggregometry. Five different agonists were selected according to the guidelines of the SSC/ISTH [75] and shipped to 15 specialized laboratories, which used two different types of light transmission aggregometers [78]. Three sets of agonists were chosen to simulate a healthy subject and inherited or acquired platelet function disorders. This trial showed very consistent data for the maximum percentage of aggregation induced by the same agonists and concentrations, demonstrating the feasibility of agonist shipment for an inter-laboratory survey of LTA. However, this trial also confirmed the results of other international surveys in that there is still a high variability in laboratory-internal agonists in terms of reagent type, concentration, and pathological cut-off values, which significantly limits the exchange of LTA results between different clinical centers [79,80,81]. The automatization of LTA has been developed to increase standardized operation, which was reviewed by Le Blanc et al. [82]. 

LTA measurements with a normal platelet count (150–600 × 10^9^/L) in PRP have been shown to be stable. Low platelet counts of less than 150 × 10^9^/L and at least ≤100 × 10^9^/L in PRP are insensitive for the valid evaluation of platelet aggregation defects [75]. Compared to whole blood assays, the platelet count here is relevant in PRP and not in whole blood, which often allows for the extension of LTA application to patients with mild and even moderate thrombocytopenia. Optional control samples from healthy donors with comparably low platelet counts adjusted with a buffer and not with PPP (>100–<150 × 10^9^/L) [50,75] and that are measured within the same day of analysis may serve as intra-laboratory references. For platelet samples from patients with macrothrombocytopenia, PRP can be generated by very low centrifugation force or by sedimentation. Here, the platelet mass has to be adjusted in control samples. 

Normal platelet aggregation responses to physiological platelet agonists—with the exception of ristocetin, which induces platelet agglutination by the VWF-mediated crosslinking of the GPIb/V/IX receptor complex—are indicative of biallelic BSS when observed with macrothrombocytopenia and when VWD could be excluded. Conversely, platelets with platelet-type VWD show increased agglutination responses induced by a subthreshold concentration of ristocetin (≤0.6 mg/mL) similar to type 2B VWD. Platelet–plasma exchange experiments with blood from patient and healthy donors are recommended to discriminate these two bleeding disorders. The differential impairment of agonist-induced platelet aggregation has been shown to be indicative of further hereditary thrombocytopenias associated with platelet function defects, including WAS, GPS, *GFI1*-related thrombocytopenia, ARC syndrome, and Stormorken syndrome [5,40,77]. Reversible aggregation characterized by an unstable second wave may be indicative for δ-granule or TxA_2_ release disorders when the agonist concentrations are titrated in a standardized manner. Notably, LTA alone is not sensitive enough for a definite diagnosis of platelet function disorders associated with thrombocytopenia, especially for mild forms such as storage pool deficiencies [83], and it therefore needs to be complemented by other specific tests of platelet phenotyping and function.

### 5.2. Lumi-Aggregometry in Platelet-Rich Plasma

Lumi-aggregometry is a further development of light transmission (PRP) and impedance-based aggregometry (whole blood) for the simultaneous detection of agonist-induced platelet aggregation and the release of ADP/ATP from δ-granules. The luciferase-catalyzed conversion of luciferin in the presence of ATP generates bioluminescence, which is detected and quantified. ADP is measured by its conversion to ATP catalyzed by pyruvate kinase in the presence of phosphoenolpyruvate. This approach can be performed in anticoagulated whole blood, but as described for other whole blood tests, analysis in PRP provides platelet-specific results [84]. Similar to LTA, lumi-aggregometry in PRP requires time and expertise for the generation of PRP and PPP. In contrast to LTA, lumi-aggregometry clearly indicates the impairment of platelet δ-granule release capacity in vitro [83] but shows a higher variability and less reproducibility than LTA [85]. This might be due to additional variables regarding the preparation and incubation of the luciferin/luciferase reagent. The secretion of δ-granules is also triggered by integrin αIIbβ3 outside-in signaling, thereby facilitating the irreversible aggregation response [86]. Thus, it should be considered that impaired platelet aggregation may affect ATP release from δ-granules in this assay. A further limitation is that δ-SPD cannot be distinguished from granule release defects (primary secretion defects), which are commonly caused by impaired signal transduction. Therefore, complementary assays have to be performed, either to validate a δ-SPD by transmission electron microscopy, the bioluminescence-/HPLC-based quantification of ATP in lysates of resting platelets, and the flow cytometric quantitation of mepacrine uptake in resting platelets (Section 3.5 and Section 5.3) or to confirm a δ-granule secretion defect by the same assays using lysates and intact platelets treated by strong agonists. The advantages and limitations for the analysis of thrombocytopenic samples are similar as described for LTA. The intra-laboratory standardization of operation and establishment of normal reference ranges are therefore also essential for LTA and lumi-aggregometry. 

### 5.3. Flow Cytometry (Whole Blood, Platelet-Rich Plasma) 

**Figure 2:**
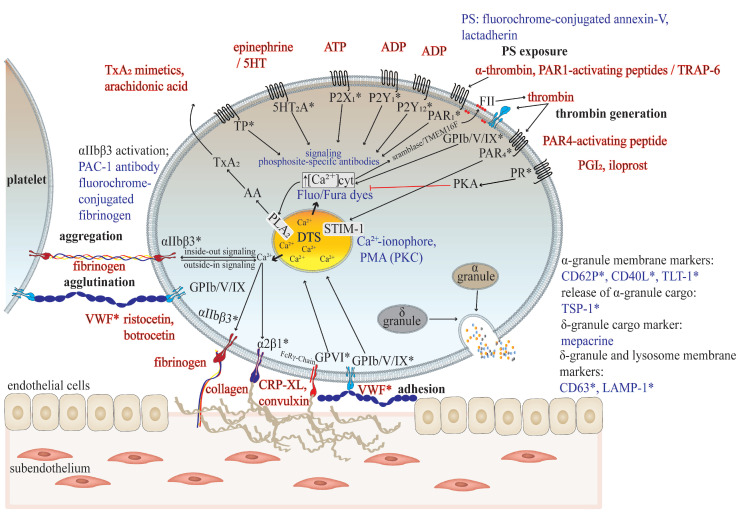
Flow cytometric analysis of the human platelet phenotype ex vivo and functional capacity in vitro in thrombocytopenia. Flow cytometry allows for a comprehensive analysis of common platelet phenotype and function defects in samples of whole blood and platelet-rich plasma, even with a very low platelet count. Commonly observed platelet granule defects (e.g., storage pool deficiency and primary secretion defects) in inherited and acquired thrombocytopenias can be detected by the surface expression of granule membrane markers stained by fluorochrome-conjugated antibodies (*) or by the antibody staining of the α-granule cargo protein TSP-1 and δ-granule cargo molecules ADP and ATP with mepacrine. Quantitative defects in platelet surface receptors, as prominently observed in acquired thrombocytopenias, are detected by staining with fluorochrome-conjugated antibodies (*), too. The use of different platelet agonists enables the detection of activated integrin αIIbβ3 (antibody PAC-1 (*) or the binding of fluorochrome-conjugated fibrinogen, indicative of platelet aggregation), VWF-binding (anti-VWF antibody staining (*), indicative of VWF-mediated platelet agglutination and biallelic BSS, platelet type VWD, and acquired VWD), and the exocytosis of granules by staining of granule membrane proteins with fluorochrome-conjugated antibodies (*). *Antigens of platelet receptors and granule membranes detected by fluorochrome-conjugated antibodies. Fluorochrome-conjugated annexin-V or lactadherin is used to quantify anionic phospholipid exposure ex vivo or in response to agonists, which determines the recruitment of coagulation factors, leading to platelet-based thrombin generation. Distinct signaling defects are detected by phospho-specific antibodies after permeabilization of the platelet surface membrane. Phospho-specific antibodies against phosphorylated VASP at S239 detect a cytosolic increase in levels of inhibitory cAMP, leading to the activation of PKA. A defective release of Ca^2+^ ions from intracellular Ca^2+^ stores (e.g., dense tubular system (DTS)) can be detected by Ca^2+^-sensitive fluorescent Fluo and Fura dyes. VWF: Von Willebrand factor; cAMP: cyclic adenosine monophosphate; ADP: adenosine diphosphate; CRP-XL: cross-linked collagen-related peptide; PAR: proteinase-activated receptor; PKA: protein kinase A; TxA_2_: thromboxane A_2_; TP: TxA_2_ receptor; AA: arachidonic acid; PLA_2_: phospholipase A_2_; PKA: protein kinase A; STIM-1: stromal interaction molecule-1; PGI_2_: prostacyclin; DTS: dense tubular system; GP: glycoprotein; PS: phosphatidylserine; TRAP-6: thrombin receptor activating peptide-6; TLT-1: trem-like transcript-1; TMEM16F: transmembrane protein 16F; TSP-1: thrombospondin-1; LAMP-1: lysosome-associated membrane glycoprotein-1; PMA: phorbol 12-myristate 13-acetate; VASP: vasodilator-stimulated phosphoprotein; PKC: protein kinase C. PKA and PKC are some examples of protein kinases, but there are many more protein kinases involved.

Flow cytometry is recommended to be used for first-line screening assays, as well as for the evaluation of specific defects in platelet phenotype and function attributes [3,87,88]. Flow cytometry represents the standard technique to quantify receptors and distinct platelet activation markers on the surface of platelets in citrated whole blood for the evaluation of quantitative receptor defects and increased platelet activation status in vivo, including the shedding of surface membrane receptors. The in vitro assessment of platelet reactivity in response to a variety of agonists (see Section 5.1, with the exception of the use of commercially available collagen types with large fibrils), especially in low amounts of whole blood, demonstrates further strengths [43,89]. A large spectrum of different fluorochrome-conjugated monoclonal antibodies, proteins, and fluorescent dyes are commercially available, which allow for a comprehensive phenotyping of single platelets and platelet populations [89,90]. The tracking of intracellular Ca^2+^ release and protein phosphorylation by using phosphosite-specific antibodies are powerful tools to evaluate signal transduction defects in platelets [91,92]. Imaging flow cytometry and mass cytometry are promising new flow cytometric developments of flow cytometry to identify platelet subpopulations [40,93]. Though there is a high variability of protocols and standard operation procedures (SOPs) between different laboratories, intra-laboratory standardization, the validation of assays, and laboratory internal reference ranges are essential and need to be established in diagnosis laboratories. Advanced protocols for platelet antibody labeling and fixation in whole blood enable the remote platelet function testing of shipped blood samples [94]. Flow cytometry-based single cell analysis provides platelet assays, which are largely independent of platelet count and thereby feasible in blood from patients even with severe thrombocytopenia [95,96]. However, a recent study indicated that a platelet count of ≤10 × 10^9^/mL might influence in vitro platelet activation assays due to the platelet count-related decreased release of ADP, which serves as important amplifier of platelet activation [97]. Figure 2 gives an overview of important platelet phenotype and activation markers, which are determined by flow cytometry for the diagnostic purposes of platelet function defects potentially associated with thrombocytopenia.

**Figure 3:**
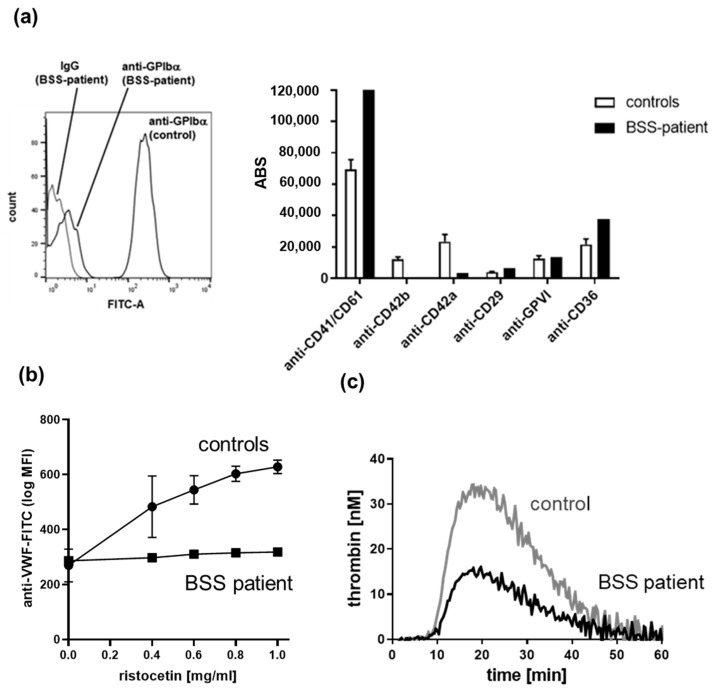
Platelet phenotype and function analysis of a patient with biallelic Bernard–Soulier syndrome (BSS) by flow cytometry and calibrated automated thrombography. (**a**) Representative flow cytometry histogram of platelets labelled with anti-GPIbα antibody SZ2-fluorescein isothiocyanate (FITC) from a donor with GPIbα-deficiency (biallelic Bernard-Soulier syndrome (BSS)) in comparison to platelets from a day control and to platelets labelled with immunoglobulin G1 (IgG1)-FITC as negative control and flow cytometric detection of antigen binding sites (ABSs) of fluorochrome-labelled antibodies against major receptors on platelets in citrated whole blood compared to healthy controls (n = 10, means ± SD). (**b**) Flow cytometric analysis of ristocetin-induced VWF-binding (labeling with anti-VWF-FITC antibody) to platelets from BSS patient compared to controls (n = 10, means ± SD). (**c**) Thrombogram (calibrated automated thrombography) of thrombin-induced thrombin generation in platelet-rich plasma from a BSS patient compared to a day control with adjusted platelet mass. MFI: mean fluorescence intensity.

The flow cytometric quantification of antigen binding sites of fluorochrome-conjugated monoclonal antibodies against platelet receptors is important to identify inherited platelet receptor defects [98], as demonstrated for the detected loss of the platelet VWF receptor complex GPIb/V/IX in a patient with biallelic BSS and a platelet count of 21 × 10^9^/L and a mean platelet volume of >13 fl (Figure 3a). The absence or severe impairment of VWF binding to platelets induced by the antibiotic ristocetin confirmed the typical functional platelet defect in BSS, especially when LTA could not be applied due to severe thrombocytopenia (Figure 3b).

Notably, in the case of macrothrombocytopenias, platelets have to be gated among their size properties to enable comparisons with control references from healthy controls. Several studies have demonstrated the clinical utility of flow cytometry for the evaluation of platelet dysfunction in acquired thrombocytopenias, such as ITP [99,100] and hematological malignancies [9,101].

**Figure 4:**
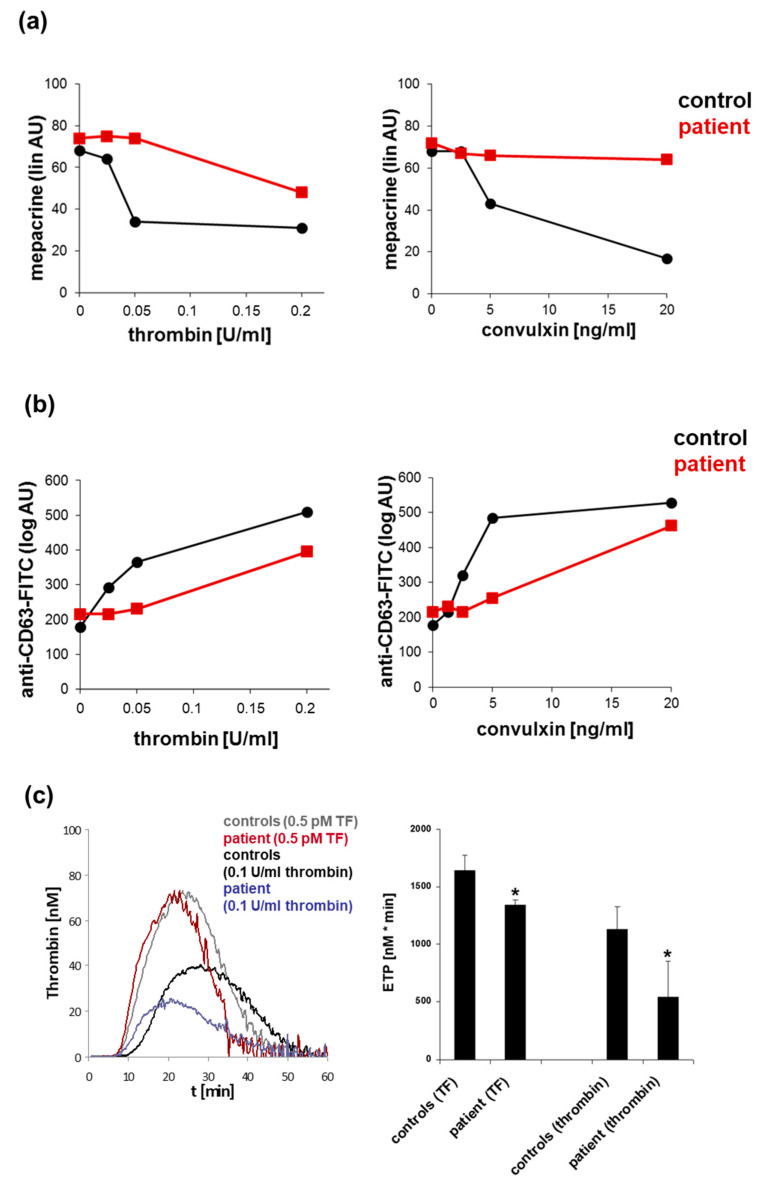
Platelet function analysis of a patient with the May–Hegglin anomaly (pathogenic *MYH9* variant E1841K) by flow cytometry and calibrated automated thrombography. (**a**) Flow cytometric analysis of mepacrine uptake in resting patient platelets and thrombin- or convulxin (GPVI-agonist)-induced mepacrine release compared to a day control. (**b**) Flow cytometric analysis δ-granule/lysosome exocytosis expressed as CD63 surface expression of patient and day control platelets in response to increasing concentrations of thrombin and convulxin, respectively. (**c**) Thrombogram (calibrated automated thrombography) and quantitation of the endogenous thrombin potential (ETP) of tissue factor (TF) or thrombin-stimulated patient and control platelets in platelet-rich plasma; three replicates; n = 3; * *p* < 0.05 versus corresponding controls. AU: arbitrary units.

Distinct flow cytometric platelet activation assays are recommended for use in buffer-diluted PRP when the detection of distinct fluorescent markers may be influenced by the hemoglobin of red blood cells, or the determination of platelet activation responses should be specifically induced by agonists without potential interference with red blood cells and leukocytes. The flow cytometric assessment of the agonist-induced release/exocytosis of platelet granules is important to complement the diagnosis of SPD. Platelets from patients with the inherited α-SPD GPS show impaired or lacking P-selectin surface expression and TSP-1 binding induced by several platelet agonists [102]. The mepacrine assay allows one to discriminate between platelet δ-SPD and a δ-granule release defect. In resting platelets, mepacrine is loaded to the δ-granules by specific binding to adenosine nucleotides such as ADP or ATP. The decreased uptake of mepacrine in resting platelets is indicative of a δ-SPD with a moderate specificity and sensitivity [103,104]. Interestingly, we observed that platelets from a patient with the *MYH*-9-related thrombocytopenia May–Hegglin anomaly, caused by the frequent *MYH9* mutation E1841K in the C-terminal exon 38 encoding for the tail part of non-muscle myosin heavy chain-IIA, showed normal mepacrine uptake but impaired release in response to increasing concentrations of thrombin and the GPVI agonist convulxin. These data were confirmed by the impaired surface expression of the δ-granule/lysosome membrane marker CD63 (Figure 1 and Figure 4a,b, Table S1). A similar release defect was observed for the α-granules expressed by impaired P-selectin surface expression, whereas the thrombin- and convulxin-induced activation of the integrin αIIbβ3 was normal [23].

This granule release defect may explain the moderated bleeding symptoms of the patient since birth, as his platelet mass was found to be relatively normal and only distributed to a smaller number of giant platelets (35–60 × 10^9^/L, variable MPV > 12 fl). Thus, the flow cytometric application of a panel of activation markers offers a comprehensive evaluation of platelet function defects in inherited and acquired thrombocytopenias.

### 5.4. Platelet-Based Thrombin Generation Tests (Platelet-Rich Plasma)

Activated platelets significantly contribute to the amplification of thrombin generation, which is essential for thrombus stabilization and for crosstalk between platelets, leukocytes, and endothelial cells [105,106] (Figure 1 and Figure 2). Platelet-dependent thrombin generation tests are offered as commercially available techniques such as the frequently used calibrated automated thrombography (CAT). Active recombinant tissue factor (TF) serves as trigger in the presence of a high Ca^2+^ concentration to induce a strong activation response of platelets in PRP (platelet count adjusted to 150 × 10^9^/L with PPP), leading to the exposure of anionic phospholipids and the subsequent formation of the tenase and prothrombinase complex. This assay allows for the continuous monitoring of fluorescence traces (which are generated through the cleavage of a fluorogenic peptide substrate by thrombin and which directly reflect the in vitro capacity of thrombin generation by platelets) over time in a 96-well plate format by a fluorescence reader [107]. This device complements flow cytometric analysis of platelet procoagulant activity for the identification of causative platelet dysfunction in platelet-based coagulation and secondary hemostasis. A recent review by Panova-Noeva, van der Meijden, and Ten Cate gave a comprehensive overview about clinical applications, pitfalls, and uncertainties of thrombin generation tests performed with PRP [108].

The knowledge about exclusive defects of platelet procoagulant activity is limited, as observed for the very rare inherited platelet function disorder Scott syndrome caused by pathogenic variants in *ANO6*, which is not usually associated with thrombocytopenia [109]. Indeed, impaired thrombin generation can be also affected by defective primary platelet responses, e.g., deficiency or decreased levels of integrin αIIbβ3 or ADP signaling [110,111]. Using thrombin as direct platelet agonist in the CAT assay, we recently confirmed impaired thrombin generation, expressed by decreased thrombin peak and endogenous thrombin potential (ETP), in PRP from a patient with δ-SPD characterized by decreased release of the feedback agonists ADP and ATP [112]. Similar results were observed for platelets from a biallelic BBS patient, when the platelet mass was adjusted in a control platelet sample from a healthy donor [112] (Figure 3c). This phenomenon can be explained by the impaired binding of thrombin to the GPIb/V/IX complex, which serves as co-receptor for PAR-mediated thrombin signaling [113]. Interestingly, we detected a decreased ETP in PRP from the already described patient with a May–Hegglin anomaly (macrothrombocytopenia, but normal platelet mass) who showed a primary secretion defect of platelet α- and δ-granules (Figure 4c). It is likely that the impaired thrombin generation resulted from a secondary granule secretion defect. Therefore, this test is very helpful to evaluate the impact of primary platelet function defects on thrombin generation and coagulation, but this technique also requires intra- and inter-laboratory standardization [114]. Samples from patients with mild-to-moderate thrombocytopenia allow for a valid analysis in a platelet count range of 50–150 × 10^9^/L in PRP [115], though preferably in direct comparison to control samples with adjusted platelet count/mass (Figure 3c and Figure 4c).

### 5.5. Microfluidics (Whole Blood)

Flow chamber devices are used to measure platelet adhesion, aggregation and thrombus formation, embolization on subendothelial matrix and plasma proteins under controlled arterial or venous shear stress, and non-coagulant or coagulant conditions in anticoagulated whole blood by advanced bright-field and fluorescence microscopy [116,117]. In comparison to the whole blood PFA and cone and plate(let) analyzer systems, parallel flow chambers have been further developed for systems biology approaches to assess in real-time multiple parameters in an integrated and time-resolving manner by multicolor imaging. Different fluorochrome-conjugated antibodies, proteins, and fluorescent dyes, which are also used for flow cytometry (Figure 2), enable the simultaneous detection of platelet activation markers, e.g., integrin αIIbβ3 activation, P-selectin surface expression, the exposure of anionic phospholipids (e.g., phosphatidylserine), intraplatelet Ca^2+^ release, and the generation of fibrin within a forming platelet aggregate/thrombus [118,119]. Multi-parameter and multi-microspot-based flow assays were recently used for the comprehensive characterization of platelet dysfunction and impaired thrombus formation from patients with inherited platelet function disorders, including the inherited thrombocytopenias GPS, May–Hegglin anomaly, and Stormorken syndrome [120,121]. Thrombus parameters are stable from healthy blood donors within normal platelet count ranges [122], but they are sensitive to low platelet counts. The establishment of reference ranges of reconstituted whole blood samples from healthy subjects with adjusted platelet count/mass is promising to distinguish between platelet count and function-related effects on thrombus formation in thrombocytopenic blood samples. Thus far, the clinical utility of microfluidics is limited for the diagnosis of platelet function and number disorders due to the lack of standardization [116] and validation in prospective cohort studies.

The integration of cultured endothelial within microfluidic devices extends the analysis of the patho(physiological) crosstalk between platelets and the vasculature, which also includes crosstalk with leukocytes [123,124]. A recent proof-of-concept study revealed that distinct components of human umbilical vein endothelial cells (i.e., glycocalyx and thrombomodulin) delay platelet adhesion and fibrin formation on endothelial cells cultured as patches on collagen/tissue factor surfaces under flow in a “vessel-on-a-chip” system [125]. Such advances are currently restricted to research purposes.

## 6. Conclusions

Whole blood platelet function tests with POC attributes, e.g., impedance aggregometry, platelet function analyzers, cone and plate(let) analyzers, and thromboelastometry/graphy, are commonly used in clinical practice. These tests provide information about the global activity of primary and/or secondary hemostasis in vitro, which are dependent on platelet count, size, and function, as well as determinants including hematocrit, leukocyte count/function, and plasma factors. Due to their sensitivity to low platelet counts, these tests should not be used in clinical centers for the diagnosis of platelet function disorders from thrombocytopenic patients. Light transmission aggregometry and lumi-aggregometry are well-established platelet function tests in specialized platelet laboratories and have become more and more attractive to be used in clinical centers. Centrifugation steps of whole blood to obtain platelet-rich and poor plasma are necessary for the analysis of distinct platelet aggregation parameters with the option of the simultaneous quantitation of ATP/ADP release from platelet δ-granules. These tests are reliable for the screening of primary platelet functions, but they have to be complemented by specialized platelet function assays and phenotyping approaches for diagnostic utility. The analysis of platelet function in samples with a low platelet count is limited but feasible and requires a comparison with control samples from healthy donors, where the platelet count (in the case of thrombocytopenia) or platelet mass (in the case of macro-/micro-thrombocytopenia) is adjusted.

Flow cytometry represents the most powerful technique for a comprehensive characterization of the platelet phenotype and activation capacity in samples from thrombocytopenic patients. It enables the analysis of samples from patients with severe thrombocytopenia and is feasible in both whole blood and platelet-rich plasma. Advanced specialized microfluidic-based tests performed in whole blood are very valuable for the evaluation of specific platelet function parameters under flow and variable coagulant conditions. Thrombin generation assays performed with platelet-rich plasma allow for the assessment of the impact of platelet function defects on coagulation/secondary hemostasis. Nevertheless, a comparison of samples from thrombocytopenic patients with platelet count/mass-adjusted reference samples from healthy donors are necessary for all these tests to distinguish between platelet function and count effects. The predictive value of platelet function testing for the estimation of the bleeding risk of thrombocytopenic patients is promising but currently limited based on only few data from prospective clinical cohort studies.

## Data Availability

Not applicable.

## Supplementary Materials

The following are available online at https://www.mdpi.com/2077-0383/10/5/1114/s1, Table S1: Thrombocytopenia-causing genes associated with platelet function defects; Table S2: Impact of thrombocytopenia on selected point-of-care and specialized platelet function tests.
