# Supplementary material for: Platelet Phenotyping and Function Testing in Thrombocytopenia
Source: J Clin Med. 2021 Mar 7;10(5):1114. doi: 10.3390/jcm10051114 (PMC7962106; doi:10.3390/jcm10051114)
Supplement: Supplementary file 1 [file jcm-10-01114-s001.pdf]

Supplementary material:

Table S1. Thrombocytopenia-causing genes associated with platelet function defects [18–23].

Mutational effects	Gene	Gene location	Protein name	Inheritance	Disease	Degree of thrombocytopenia	Platelet size	Platelet function defects	Degree of bleeding
Megakaryopoiesis	*ETV6*	12p13.2	Transcription factor ETV6	AD	*ETV6*-related thrombocytopenia	+/++	N/L	reduced ability to spread on fibrinogen	mild
	FLI1	11q24.3	Friend leukemia integration 1 transcription factor	AR	Paris-Trousseau thrombocytopenia/Jacobsen syndrome	++	N/L	defect in platelet GPVI, GPIb/IX, GPIIb, abnormal α-granules, δ-SPD, with reduced ability of platelets to aggregate	moderate
	GATA1	Xp11.23	Erythroid transcription factor	XL	*GATA1*-related disease: X-linked thrombocytopenia (XLT) and X-linked thrombocytopenia with thalassemia (XLTT)	+++	N/L	less α-granules or α-/δ-SPD	moderate to severe
	GFI1B	9q34.13	Zinc finger protein GfI-1b	AD	*GFI1b*-related thrombocytopenia, Gray Platelet-like syndrome	+/++	L	decreased or absent α-granules, α-/δ-SPD	moderate to severe
	NBEAL2	3p21.31	Neurobeachin-like protein 2	AR	Gray platelet syndrome	++/+++	L	decrease or absence of platelet α-granules	mild to severe
	Deletions	11q23-24 (encoding FLI1)	friend leukemia integration	AD	Paris–Trousseau thrombocytopenia, Jacobsen syndrome	+++	N/L	abnormal giant α-granules in platelets, δ-SPD	moderate to severe
	ANKRD26	10p12	Ankyrin repeat domain-containing protein 26	AD	*ANKRD26*-related thrombocytopenia	++/+++	N	reduced α-granules in some patients	mild
	RUNX1	21q22.12	Runt-related transcription factor 1	AD	Familial platelet disorder with propensity to acute myelogenous leukemia (FPD/AML)	++	N/L	defects of αIIbβ3 activation and platelet aggregation and α/δ-SPD	moderate
	SLFN14	17q12	Protein SLFN14	AD	*SLFN14*-related thrombocytopenia	+/++	L	reduced number of δ-granules and defective platelet aggregation and decreased ATP secretion have been reported	moderate to severe
	SRC	20q11.23	Proto-oncogene tyrosine-protein kinase Src	AD	*SRC*-related thrombocytopenia	++	L	deficiency of platelet α-granules and abundant vacuoles	severe
	ABCG5	2p21	ATP-binding cassette sub-family G member 5	AR	Sitosterolemia with macrothrombocytopenia	+/++	L	hypertrophic and hyperplasic dysfunctional platelets due to accumulation of plant sterols in platelet membranes	mild to moderate
	ABCG8	2p22	ATP-binding cassette sub-family G member 8	AR	Sitosterolemia with macrothrombocytopenia	+/++	L	hypertrophic and hyperplasic dysfunctional platelets due to accumulation of plant sterols in platelet membranes	mild to moderate
	PTPRJ	11p11.2	Receptor-type tyrosine-protein phosphatase eta	AR	Inherited thrombocytopenia	++/+++	S	impaired platelet function due to decreases GPVI expression has been hypothesized	mild
	RBM8A	1q21	RNA-binding protein 8A	AR	Thrombocytopenia-absent radius syndrome	+	S/N	decreased numbers of δ-granules	moderate to severe
	PTPN11	12q24.13	Tyrosine-protein phosphatase non-receptor type 11	AD	Noonan syndrome	++	L	encodes SHP2 which a critical regulator of signal transduction	moderate to severe
	GALE	1p36.11	UDP-glucose 4-epimerase	AR	Inherited thrombocytopenia	+++	N/L	pale platelets	mild
	RAB27A	15q21.3	Ras-related protein Rab-27A	AR	Griscelli syndrome, type 2; GS2	+	N	reduced platelet aggregation, decreased numbers of δ-granules	mild
Platelet production and/or platelet clearance	*VIPAS39*	14q24.3	Spermatogenesis-defective protein 39 homolog	AR	Arthrogryposis, renal dysfunction, and cholestasis 2	+	N/L	leads to significant α-granule deficiencies in platelets and P-selectin is severely decreased.	our knowledge of the clinical consequences of the platelet defects is limited due to children with ARC mostly die in the first years of life
	PLAU	10q22.2	Urokinase-type plasminogen activator (u-PA)	AD	Quebec syndrome	++	N	increased u-PA levels within platelets lead to intraplatelet plasmin generation and secondary degradation of α-granule proteins	moderate to severe

GP1BA	17p13.2	Platelet glycoprotein Ib alpha chain	AR	biallelic Bernard-Soulier syndrome (BSS)	++/+++	N/L	abscence or dysfunction of the platelet glycoprotein receptor Ib/V/IX complex and reduced agglutination/aggregation response to ristocetin, reduced adhesion to VWF/collagen	moderate to severe
GP1BA	17p13.3	Platelet glycoprotein Ib alpha chain	AD	monoallelic Bernard-Soulier syndrome (BSS)	+/++	L	BSS caused by absence or dysfunction of the platelet glycoprotein receptor Ib/V/IX complex with absent or markedly reduced aggregation in response to ristocetin	mild
GP1BA	17p13.3	Platelet glycoprotein Ib alpha chain	AD	platelet type von-Willebrand disease (PT-VWD)	++/+++	N/L	spontaneous platelet aggregation in vitro	Moderate to severe
FLNA	Xq28	Filamin-A	XL	Filaminopathies A	++	L	abnormal platelet morphology, heterogeneous distribution of α-granules	Moderate
GPIBB	22q11	Platelet glycoprotein Ib beta chain	AD	Bernard– Soulier syndrome	++/+++	L	abscence or dysfunction of the platelet glycoprotein receptor Ib/V/IX complex and reduced agglutination/aggregation response to ristocetin, reduced adhesion to VWF/collagen	moderate to severe
GP9	3q21.3	Platelet glycoprotein IX	AD	Bernard–Soulier syndrome, monoallelic (type C)	+	L	abscence or dysfunction of the platelet glycoprotein receptor Ib/V/IX complex and reduced agglutination/aggregation response to ristocetin, reduced adhesion to VWF/collagen	mild
ITGA2B	17q21	Integrin alpha-IIb	AD	ITGA2B/ITGB3-related thrombocytopenia	+/++	N	mild aggregation defects	mild to moderate
ITGB3	17q21	Integrin beta-3	AD	ITGA2B/ITGB3-related thrombocytopenia	+/++	N	mild aggregation defects	mild to moderate
MYH9	22q12.3	Myosin-9	AD	*MYH9*-related disease (MYH9-RD)	+/++	L/G	case report [23]granule release defect and impaired thrombin generation detected for patient with May-Hegglin anomaly (pathogenic *MYH9* variant E1841K)	mild to severe
PRKACG	9q21.11	cAMP-dependent protein kinase catalytic subunit gamma	AR	*PRKACG*-related thrombocytopenia	+++	L/G	encodes the gamma isoform of the catalytic subunit of PKA, therefore its mutation causes a defect in platelet activation	severe
TRPM7	15q21.2	Transient receptor potential cation channel subfamily M member 7	AD	*TRPM7*-related thrombocytopenia	++	L	abberant distribution of granules, increased number and anarchic organization of microtubules	mild
TPM4	19p13.12-p13.11	Tropomyosin alpha-4 chain	AD	Tropomyosin 4-related thrombocytopenia	+	L	mild effect on platelet function	mild
STIM1	11p15.4	Stromal interaction molecule 1	AD	Stormorken syndrome	+/++	N/L	platelet dysfunction and reduced aggregation to ADP and collagen, increased intracellular Ca^{2+} levels, activated integrin αIIbβ3, increased PS exposure, impaired α-granule release and clot retraction (impaired integrin αIIbβ3 outside-in signaling)	mild to severe
GNE	9p13.3	Bifunctional UDP-N-acetylglucosamine 2-epimerase/N-acetylmannosamine kinase	AR	*GNE* myopathy with congenital thrombocytopenia	+	N	defect in GPIb/IX receptors and changes in surface sialylation	mild
FYB	5p13.1	FYN-binding protein 1	AR	*FYB*-related thrombocytopenia	++/+++	S/N	platelets showed reduced pseudopodium formation, increased level of activated platelets	mild to moderate
WAS	Xp11.23	Wiskott-Aldrich syndrome protein	XL	Wiskott-Aldrich syndrome, X-linked thrombocytopenia (XLT)	+++	S/N	decreased aggregation and secretion, decreased numbers of δ-granules, impaired clot retraction (defect in integrin αIIbβ3 outside-in signaling)	severe

Degree of thrombocytopenia: +, >100–<150 × 10^9 platelets/L; ++, 50–100 × 10^9 platelets/L; +++, <50 × 10^9 platelets/L. Inheritance: AR; autosomal recessive, AD; autosomal dominant, XL; X-linked. Platelet size: S; small, N; normal, L; large, G; giant; SPD: storage pool deficiency. Website: www.omim.org; www.orpha.net; www.omim.org; www.isth.org; www.malacards.org; www.uniprot.org.

Table S2. Impact of thrombocytopenia on selected point-of-care-related and specialized platelet function tests [40,89].

Type	Method	Sample	Method principle	Platelet dysfunction sensitivity	Impact of thrombocytopenia
**Point-of-care-related platelet function tests**	Multiple Electrode Aggregometry (MEA, Multiplate® Analyzer)	WB	impedance-based detection of electrode coating by aggregated platelets over time	monitoring anti-platelet therapy response	sensitive to thrombocytopenia, control samples with adjusted platelet count/mass recommended, not recommended for routine practice
	Platelet Function Analyzer (PFA-100®, INNOVANCE® PFA-200)	WB	occlusion of an aperture coated with collagen/epinephrine or collagen/ADP at high arterial shear rate	only severe platelet defects (e.g., BSS)	not recommended for platelet count < 100 × 10^9/L
	Cone and Plate(let) Analyzer (CPA, Impact-R™)	WB	shear-induced platelet adhesion/aggregation VWF-immobilized polystyrene surface	moderate-severe hypo and hyperreactive platelets	sensitive to thrombocytopenia, control samples with adjusted platelet count/mass recommended
	Thromboelastography (TEG® – Platelet Mapping™) /Thromboelastometry (ROTEM®)	WB	viscoelastic-based detection of clot strength	limited studies for platelet-dependent coagulation defects	sensitive to thrombocytopenia, control samples with adjusted platelet count/mass recommended
**Specialized platelet function tests**	Light Transmission Aggregometry (LTA)	PRP	Photo-optical measurement of light transmission increase in relation to agonist-induced platelet aggregation/agglutination	limited sensitivity to δ-SPD or primary secretion defects	not recommended for platelet count ≤ 100 × 10^9/L in PRP, control samples with adjusted platelet count/mass recommended
	Lumi-Aggregometry (Chrono-Log®)	PRP	bioluminescent detection of released ATP during agonist-induced platelet aggregation	detection of storage pool and secretion defects of δ-granules but no discrimination	not recommended for platelet count ≤ 100 × 10^9/L in PRP, control samples with adjusted platelet count/mass recommended
	Flow cytometry-based tests	WB, PRP	laser-based detection of fluorescently- labelled singular platelets	detection of a panel of platelet activation and signaling markers ex vivo and in response to agonists (Figure 2)	recommended technique for mild, moderate and severe thrombocytopenia, potentially sensitive for platelet count ≤ 10 × 10^9/L
	Thrombin Generation Assay (TGA) / Calibrated Automated Thrombino - graphy (CAT)	PRP	fluorescence-based kinetic assay of thrombin-mediated substrate cleavage triggered by active tissue factor	sensitive for intrinsic platelet-dependent coagulation defects and moderate-severe platelet dysfunction	not recommended for platelet count < 50 × 10^9/L in PRP, control samples with adjusted platelet count/mass recommended
	Microfluidics	WB	light/fluorescence microscopy-based simultaneous multi-parameter assessment of shear-dependent platelet adhesion, aggregation, coagulation and thrombus formation on extracellular matrix proteins or on endothelial cells	Systems biology approach for the analysis of platelet dysfunction	sensitive to thrombocytopenia, control samples with adjusted platelet count/mass recommended

WB; whole blood, PRP; platelet-rich plasma, ADP; adenosine diphosphate, BSS; Bernard-Soulier syndrome, SPD; storage pool deficiency, VWF; von Willebrand factor, ATP; adenosine triphosphate, PFA; platelet function analyzer, CPA; cone and platelet analyzer, MEA; multiple electrode aggregometry, TEG; thromboelastography, ROTEM; rotational thromboelastometry, TGA; thrombin generation assay, CAT; calibrated automated thrombinography.
